# Cocrystallization of Antifungal Compounds Mediated by Halogen Bonding

**DOI:** 10.1021/acs.cgd.3c00067

**Published:** 2023-03-08

**Authors:** Mónica Benito, Antonio Frontera, Elies Molins

**Affiliations:** † Institut de Ciència de Materials de Barcelona (ICMAB-CSIC), Campus UAB, 08193 Bellaterra, Spain; ‡ Departament de Química, Universitat de les Illes Balears, Ctra. Valldemosa km 7.5, E-07122 Palma de Mallorca, Spain

## Abstract

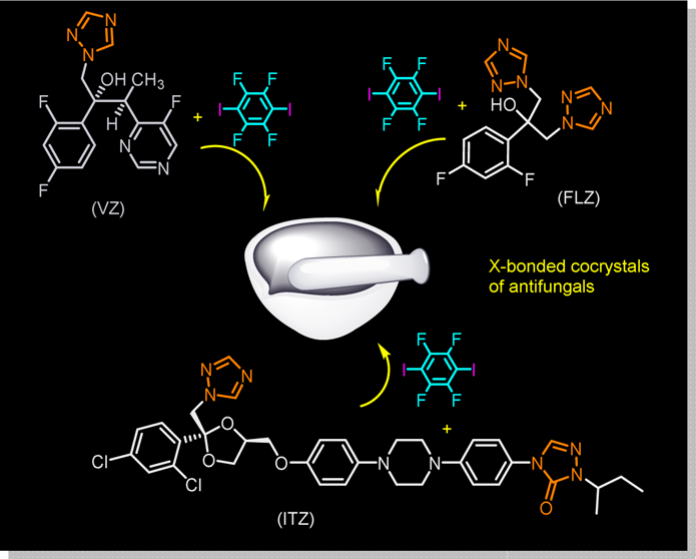

The application of halogen bonding in pharmaceutical chemistry remains a challenge. In this work, novel halogen-bonded cocrystals based on azole antifungal active pharmaceutical ingredients (APIs) and the ditopic molecule 1,4-diiodotetrafluorobenzene are reported. Their crystal structural features, spectroscopic properties, and thermal stability were studied. The components are bound through I···N from the triazole moieties present in all of the compounds. The molecular electrostatic potential (MEP) surfaces and quantum theory of atoms in molecules (QTAIM) calculations are used to rationalize the presence of hydrogen and halogen bonds in the resulting structures and their energetic analysis. The relative halogen bond ability of the different groups of voriconazole, fluconazole, and itraconazole was analyzed using MEP surfaces, demonstrating this approach to be an interesting tool to predict halogen-bonding preferences.

## Abstract 2

New halogen-bonded cocrystals of azole antifungal APIs with the ditopic 1,4-diiodotetrafluorobenzene were synthesized. Their crystal structures, intermolecular interactions, and thermal stability were studied with combined DFT calculations.

## 1 Introduction

Noncovalent interactions, mostly hydrogen bonding, are responsible for many tridimensional networks in the solid state. Other supramolecular interactions include halogen bonds, being also a highly directional and linear interaction.^1,2^ Exploration of halogen bonding (HaB) through cocrystallization with organic molecules is well established. However, the use of active pharmaceutical ingredients (APIs) in this playground is still scarce. Halogen atoms can be present in the API backbone because of the need to tune their lipophilic behavior when crossing lipid membranes and to increase their activity before drug decomposition in the body. Thus, halogen···halogen contacts and halogen bonds can also be present and responsible for the supramolecular network in molecular solids. All of these interactions can be exploited in the search for new functional materials and applications.^3,4^

Cocrystals are molecular solids containing at least two neutral solid compounds.^5^ A few halogen-bonded pharmaceutical cocrystals have been described in the literature.^6−12^ We have recently reported several halogen-bonded cocrystals of nucleobase compounds as adenine and uracil derivatives, including an antineoplastic substance.^13,14^ Now, in this work, we expand this strategy to a different class of molecules, antifungal APIs, selectively used to eliminate infections caused by fungal pathogens. We have studied the feasibility of azole antifungals for halogen bonding. With this purpose in mind, first, different azole antifungals were carefully considered taking into account the presence of multiple functional groups (alcohol, triazole, pyrimidine, triazolone, dioxolane, etc.) in those molecules. Finally, the following three antifungal molecules voriconazole (**VZ**), fluconazole (**FLZ**), and itraconazole (**ITZ**) were selected as model compounds.

**Scheme 1:**
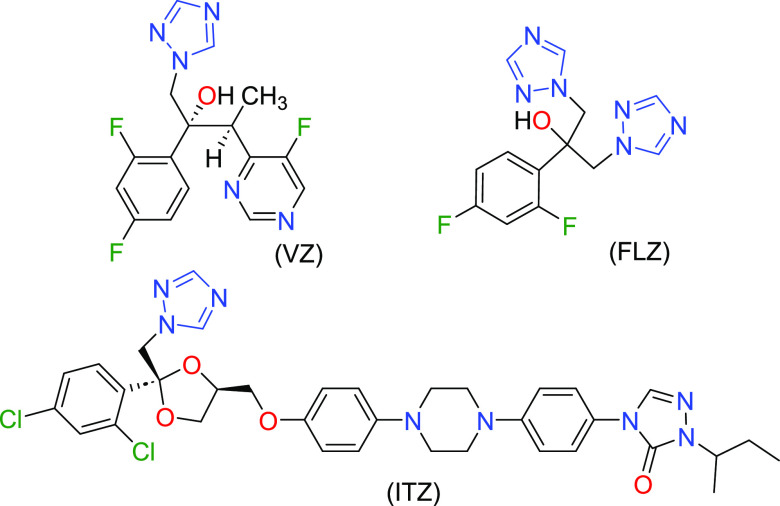
Molecular Structures of Antifungal Compounds Used in This Work

All three APIs contain halogen atoms (F or Cl) in their skeleton as well as one or two triazole rings (see Scheme 1). Except for voriconazole, the other two molecules (fluconazole and itraconazole) show polymorphism, and several solvates, and salts or hydrogen-bonded cocrystals have been described in the literature.^15−32^

In 2013, a cocrystal of itraconazole with succinic acid was revisited and a weak halogen bond was identified between C–Cl···O from the triazolone moiety and a chlorine atom.^21^ Herein, inspired by that work, the ditopic coformer 1,4-diiodotetrafluorobenzene (**DITFB**), in which iodine atoms are activated by the presence of fluorines,^11^ has been used to explore the preparation of multicomponent solids and understand the possible competition between hydrogen and halogen bonds in these pharmaceutical active molecules.

The new multicomponent solids were prepared by solvent-assisted grinding and by slow evaporation crystallization. Later, characterization by X-ray diffraction (powder and single crystal), infrared spectroscopy, and thermal methods was performed. The use of DFT and QTAIM has allowed us to measure the strength of the halogen bonds besides other interesting intramolecular H-bonds. Moreover, the MEP surface analysis allows us to identify those groups that are better halogen bond acceptors and rationalize the cocrystal formation.^33^

## 2 Experimental Section

### 2.1 Materials and Methods

Voriconazole (**VZ**), fluconazole (**FLZ**, Form II), and itraconazole (**ITZ**, Form I) were kindly donated by local pharmaceutical companies and used without further purification. 1,4-Diiodotetrafluorobenzene (**DIFTB**) was purchased from Cymit Química S.L. (Barcelona, Spain). All of the solvents used in the present work were analytical grade and were obtained from local suppliers.

### 2.2 Synthesis of Cocrystals

#### 2.2.1 **VZ**·**DITFB**

Voriconazole (100.0 mg, 0.286 mmol) and 1,4-diiodotetrafluorobenzene (115.3 mg, 0.287 mmol) were ground together with three drops of methanol in a Retsch MM400 mixer mill using a 10 mL agate jar with two 5 mm agate balls at 30 Hz for 30 min. The resulting solid was recovered and analyzed by PXRD. Slow evaporation of this solid in methanol afforded single crystals.

#### 2.2.2 **(FLZ)_2_**·**DITFB**

Fluconazole (100.08 mg, 0.327 mmol) and 1,4-diiodotetrafluorobenzene (65.64 mg, 0.163 mmol) were ground together with three drops of methanol in a Retsch MM400 mixer mill using a 10 mL agate jar with two 5 mm agate balls at 30 Hz for 30 min. The resulting solid was recovered and analyzed by PXRD. Suitable single crystals were obtained by slow evaporation of this solid in a methanol solution.

#### 2.2.3 **(ITZ)_2_**·**DITFB**

Suitable single crystals of this phase were obtained by slow evaporation of a mixture of precursors (1:1 molar ratio) in methanol. The crystal resolution afforded a new compound with a molar stoichiometry 2:1 for the **ITZ-DITFB** system.

#### 2.2.4 **ITZ**·**(DITFB)_2_**

Itraconazole (75.09 mg, 0.106 mmol) and 1,4-diiodotetrafluorobenzene (85.55 mg, 0.213 mmol) were ground together with three drops of methanol in a Retsch MM400 mixer mill using a 10 mL agate jar with two 5 mm agate balls at 25 Hz for 30 min. The resulting solid was recovered and analyzed by PXRD.

### 2.3 X-ray Diffraction Studies

A Siemens D5000 powder diffractometer was used for all X-ray powder (XRPD) measurements with experimental parameters as follows: Cu Kα radiation (λ = 1.5418 Å), scanning interval 2–50° 2θ, step size 0.02°, and exposure time 1 s per step. A single crystal of compound **VZ**·**DITFB** was selected for single-crystal X-ray diffraction experiments and mounted at the tip of a nylon CryoLoop on a Bruker D8 QUEST ECO (Photon II detector) diffractometer using graphite-monochromated Mo Kα radiation (λ = 0.71073 Å). Single crystals of compounds **(FLZ)_2_**·**DITFB** and **(ITZ)_2_**·**DITFB** were selected for single-crystal X-ray diffraction experiments and mounted at the tip of a nylon CryoLoop on a Bruker Apex-II CCD diffractometer using graphite-monochromated Mo Kα radiation (λ = 0.71073 Å). Crystallographic data for **(FLZ)_2_**·**DITFB** and **(ITZ)_2_**·**DITFB** were collected at 294(2) and 298(2) K, respectively. Data reduction was performed using SAINT v6.45A and SORTAV in the diffractometer package.^34^ Data were corrected for Lorentz and polarization effects and for absorption by SADABS.^35^ The structural resolution procedure was made using SHELXT.^36^ Non-hydrogen atoms were refined anisotropically. Hydrogen atoms were introduced in calculated positions and refined riding on their parent atoms. Selected crystal and data collection parameters are reported in the corresponding Table 1.

**Table 1:** Crystallographic Data and Structural Refinement Parameters for Cocrystals.

compound	**VZ**·**DITFB**	**(FLZ)_2_**·**DITFB**	**(ITZ)_2_**·**DITFB**
empirical formula	C_22_H_14_N_5_OF_7_I_2_	C_32_H_24_F_8_I_2_N_12_O_2_	C_38_H_38_Cl_2_F_2_IN_8_O_4_
formula weight	751.18	1014.412	906.56
temperature (K)	100(2)	294(2)	298(2)
crystal system	monoclinic	triclinic	triclinic
Space group	*P*2_1_	*P*-1	*P*-1
*a* (Å)	12.991(14)	7.6899(5)	8.424(2)
*b* (Å)	5.842(5)	11.8232(7)	10.008(2)
c (Å)	16.427(18)	11.9432(7)	25.115(6)
α (°)	90	114.1540(10)	81.240(5)
β (°)	106.94(4)	102.6170(10)	84.266(5)
γ (°)	90	96.5540 (10)	70.157(5)
*V* (Å^3^)	1193(2)	941.51(10)	1965.6(8)
*Z*	2	2	2
calc. density (Mg/m^3^)	2.092	1.789	1.532
absorption coefficient (mm^–1^)	2.719	1.758	1.011
F(000)	716	494	918
crystal size (mm^3^)	0.150 × 0.120 × 0.080	0.260 × 0.220 × 0.110	0.180 × 0.150 × 0.130
theta range for data collection (°)	2.592–28.347°	1.939–28.344°	1.643–28.435°
index ranges	–17 ≤ *h* < 17,	–10 ≤ *h* ≤ 10,	–11 ≤ *h* ≤ 11,
	–7 ≤ *k* ≤ 7,	–15 ≤ *k* ≤ 15,	–13 ≤ *k* ≤ 13,
	–21 ≤ *l* ≤ 21	–15 ≤ *l* ≤ 15	–33 ≤ *l* ≤ 33
reflections collected	19530	27535	42991
independent reflections	5904[*R*(int) = 0.0575]	4692[*R*(int) = 0.0189]	9830[*R*(int) = 0.0959]
completeness to θ max (%)	99.8%	100.0%	99.9%
max. and min. transmission	0.812–0.686	1–0.82	1–0.66
refinement method		Full-matrix least-squares on F^2^	
data/restraints/parameters	5904/7/336	4692/0/256	9830/2/498
Goodness-of-fit on F^2^	1.088	1.106	1.021
final *R* indices [*I* > 2σ(*I*)]	*R*_1_ = 0.0405,	*R*_1_ = 0.0215	*R*_1_ = 0.0864
	w*R*_2_ = 0.0547	w*R*_2_ = 0.0539	w*R*_2_ = 0.2262
*R* indices (all data)	*R*_1_ = 0.0591,	*R*_1_ = 0.0235,	*R*_1_ = 0.1675,
	w*R*_2_ = 0.0639	w*R*_2_ = 0.0555	w*R*_2_ = 0.2758
largest diff. peak and hole (e/Å^3^)	1.003 and −0.767	0.649 and −0.235	1.395 and −0.757
CCDC no.	2234082	2234081	2234080

Calculated X-ray powder patterns were obtained from single-crystal structure data using Mercury 4.3.1 software.^37^

### 2.4 Vibrational Spectroscopy

IR spectra were collected using a Jasco 4700LE spectrophotometer with attenuated total reflectance accessory at a resolution of 4.0 cm^–1^.

### 2.5 Thermal Analysis

A simultaneous thermogravimetric analysis (TGA)–differential scanning calorimetry/differential thermal analysis (heat flow DSC/DTA) system (NETZSCH-STA 449 F1 Jupiter) was used to perform thermal analysis on the solids. Samples (3–8 mg) were placed in open alumina pan and measured at a scan speed of 10 °C/min from ambient temperature to 300 °C under a N_2_ atmosphere as protective and purge gas (their respective flow velocities were 20 and 40 mL/min).

### 2.6 Theoretical Methods

The energetic analysis of the intermolecular halogen-bonding interactions was performed using the Gaussian-16 suite of programs^38^ and the PBE0-D3/def2-TZVP level of theory.^39,40^ The crystallographic coordinates were used to estimate the interactions in the solid state. The interaction energies of the halogen-bonding contacts were computed by calculating the difference between the energies of isolated monomers and their assembly. The binding energies were corrected for the basis set superposition error (BSSE) by using the Boys–Bernardi method.^41^ Bader’s “atoms in molecules” theory (QTAIM)^42^ was used to study and estimate the association energies of the intramolecular H-bonding interactions by using the AIMAll program.^43^ The molecular electrostatic potential (MEP) surfaces (isosurface 0.001 a.u.) were computed using Gaussian-16 software.^38^

To analyze the nature of interactions (attractive or repulsive) and uncover them in real space, the NCIPLOT visualization index was used. It plots the reduced density gradient (RDG) regions^44^ derived from the electronic density (ρ).^45^ The sign of the second Hessian eigenvalue (λ_2_) multiplied by the electron density (i.e., sign [λ_2_] ρ in atomic units) was employed for the identification of attractive/stabilizing (blue-green colored isosurfaces) or repulsive (yellow-red colored isosurfaces) interactions using 3D plots. The NCIplot index parameters used in this article are RGD = 0.45; ρ cutoff = 0.04 a.u.; color range: −0.04 a.u. ≤ sign(λ_2_) ρ ≤ 0.04 a.u.

## 3 Results and Discussion

### 3.1 PXRD and Spectroscopic Characterization

**Figure 1:**
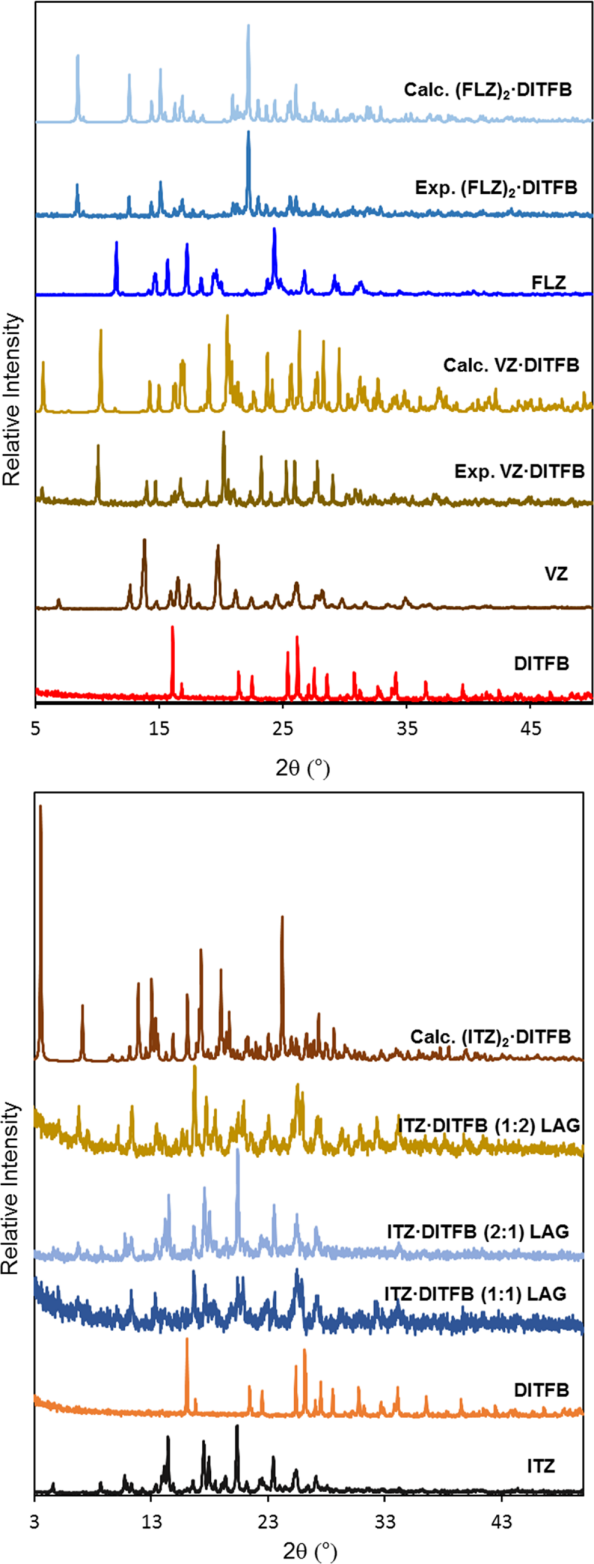
Experimental and simulated powder patterns of the new cocrystals and precursors.

Powder XRD was used to identify the formation of new crystalline phases with respect to other forms available for precursors. For **FLZ**, several polymorphs, solvates, and hydrogen-bonded cocrystals are described in the literature.^26−32^ Structurally, this is the simplest conazole of the three molecules, as two triazole rings besides a hydroxyl group are the only available functional groups in this drug. The solvent-drop grinding of **FLZ** form II and **DITFB** in 2:1 molar ratio afforded a diffractogram exhibiting a new phase (**(FLZ)_2_**·**DITFB**, Figure 1) different from the former compounds or any other solid forms previously found in a CCDC search. For other molar ratios tested (1:1 or 1:2), mixtures of the new phase and the corresponding precursor in excess were obtained.

**Figure 2:**
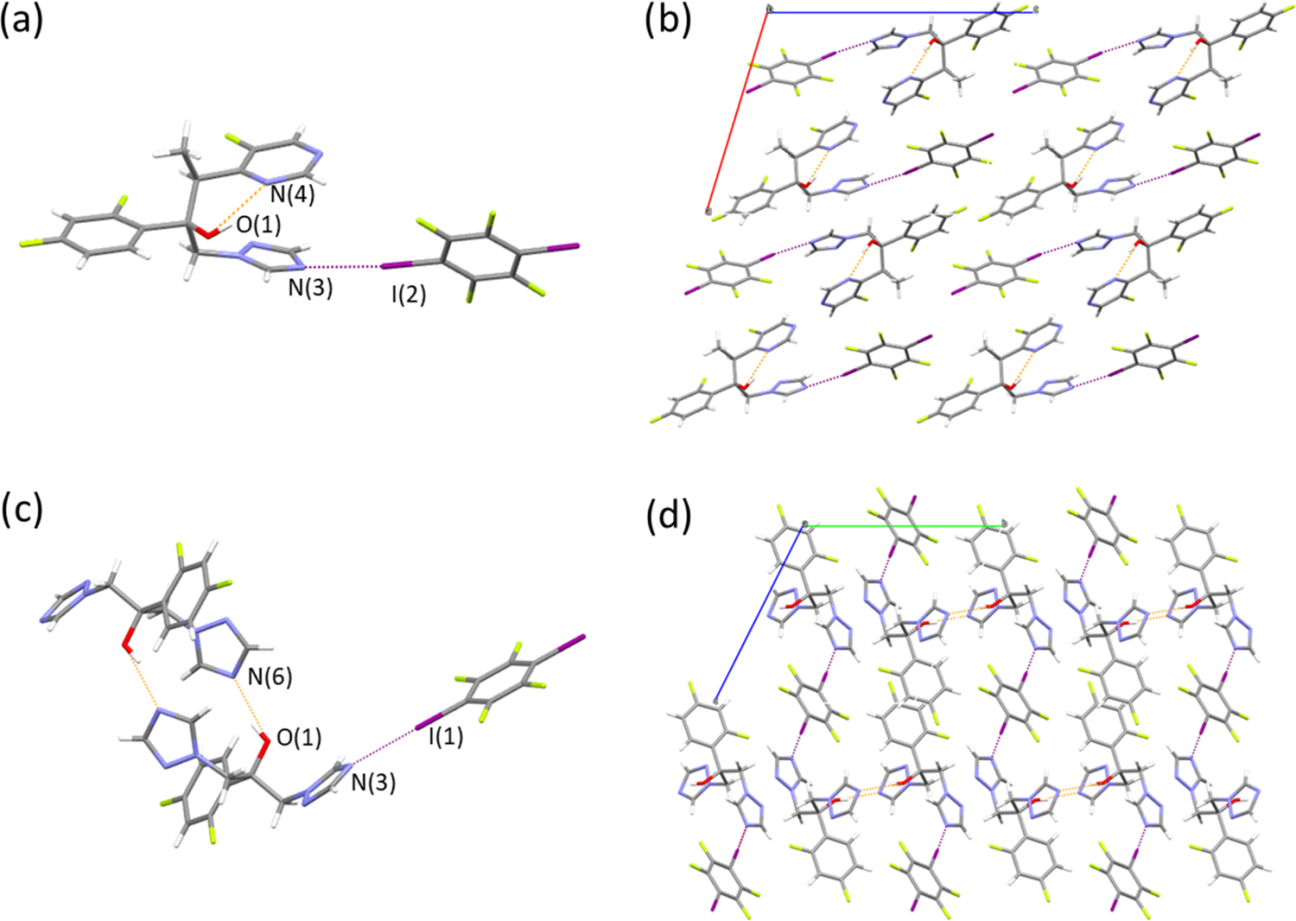
(a) **VZ**·**DITFB** cocrystal showing the intramolecular H-bond (in orange) and the Ha-bond with **DITFB** (in purple), (b) packing down *b-*axis view, (c) **(FLZ)_2_·DITFB** cocrystal showing the intermolecular H-bonds (in orange) and the Ha-bond with **DITFB** (in purple), and (d) packing down *a-*axis view.

Unlike the previous conazole, for **VZ** only one polymorphic form has been previously described in the literature, although several H-bonded cocrystals and salts have been reported.^15−19^ Moreover, with respect to **FLZ**, one triazole ring has been substituted by a fluoropyrimidine ring and a methyl group. The powder pattern of a 1:1 mixture of precursors ground with a few drops of methanol resulted in a new phase (Figure 1, cocrystal **VZ**·**DITFB**). Finally, the third antifungal tested, **ITZ**, shows several polymorphic forms and cocrystals containing different carboxylic acids or amino acids as coformers.^20−25^ This is a longer and flexible molecule with many more functional groups than **FLZ** or **VZ**. Liquid-assisted grinding (LAG) of mixtures of **ITZ** form I and **DITFB** in different molar ratios (1:1, 2:1, or 1:2) afforded powder diffractograms with low crystallinity in which new peaks were observed at 5.0, 6.7, 7.6, 10.0, 20.9, or 23.0°, although a few peaks of **ITZ** or **DITFB** could be detected. Furthermore, slow evaporation of the 1:1 solid in methanol (or in a toluene–nitromethane mixture) rendered a few days later a concomitant mixture of platelet and needle single crystals. The calculated powder pattern from these platelet crystals gave a different phase, **(ITZ)_2_**·**DITFB**, from the precursors or the one observed by grinding, with a 2:1 molar ratio composition (**ITZ** ·**DITFB** (2:1) LAG in Figure 2) and with a very characteristic peak at low 2θ values, 3.5°. Finally, by LAG, the 1:2 mixture was the most crystalline sample, and new peaks were clearly identified and assigned as a new form, namely, **ITZ**·**(DITFB)_2_**; see Figure 1.

The FTIR spectra of the three conazoles and their respective cocrystals were measured and compared to study the effect of the halogen bond formation. In Figure S1, we show the comparison for **FLZ**, **VZ**, or **ITZ**, the halogenated coformer, and the new cocrystals. The presence of the OH group was identified for the two small conazoles, **FLZ** and **VZ**, and their new solid phases. Moreover, in the case of their cocrystals, the typical vibrations for C=C or C=N from pyrimidyl and triazole rings were also followed. Thereby, a band splitting was observed in the **(FLZ)_2_**·**DITFB** cocrystal for the C=C bond with respect to **FLZ**. Moreover, in both cocrystals, shifts in the C=N (triazole) bands were observed. In the case of **ITZ** or its solid forms, the shift of the C=O vibrational mode resulted in characteristic peaks. While for the **(ITZ)_2_**·**DITFB** crystal, it appeared at 1701 cm^–1^, for the **ITZ**·**DITFB** (1:2) LAG composition, it shifted to 1672 cm^–1^. The decreasing wavenumber value with respect to pure **ITZ** (at 1693 cm^–1^) indicates a different interaction of the triazolone ring in the new form. Taking into consideration the molar ratio and the appearance of this new band, the interaction mode shown in Figure S2 is proposed. Finally, the presence of the typical bands approximately at 1463, 940, and 760 cm^–1^ for the Ha-bond donor was confirmed in all of the multicomponent solids.

### 3.2 Crystal Structure Analysis

#### 3.2.1 **VZ**·**DITFB** Cocrystal

Cocrystal **VZ**·**DITFB** crystallizes in the monoclinic space group *P*2_1_ containing one molecule of voriconazole and a molecule of the halogenated coformer in the asymmetric unit. In former voriconazole, an intramolecular hydrogen bond between the alcohol and one nitrogen from the pirimidinyl ring is found besides other H-bonds, all together responsible for the molecular arrangement observed in the crystal packing.^15^ In cocrystal **VZ**·**DITFB**, the alcohol forms the intramolecular H-bond between O(1)-H···N(4) (distance: 2.784(8) Å), although it is longer/weaker than that observed in **VZ** (refcode: CEXMAU, distance: 2.680(5) Å). An intermolecular H-bond with another voriconazole molecule through O(1)-H(1)···N(2) is also observed. Moreover, the triazole ring establishes a new HaB with the coformer by N(3)···I(2) interactions (Figure 2a). The other iodine atom does not form halogen bonds. The tridimensional packing is the result of other numerous H-bonding C–H···O, C–H···N, and C–H···F interactions (see Table S1) or F···F contacts among the fluorine atoms from voriconazole and the coformer molecules (Figure 2b).

#### 3.2.2 **(FLZ)_2_**·**DITFB** Cocrystal

This cocrystal crystallizes in the triclinic space group *P*1̅ containing two molecules of fluconazole and a molecule of **DITFB** in the asymmetric unit. Fluconazole molecules self-assemble establishing two symmetrically equivalent intermolecular H-bonds between one of the triazole’s N-atom and the alcohol (N(6)···H-O(1) interactions, distance: 2.773(2) Å), which are shorter than the equivalent H-bond observed in **FLZ** form II (refcode IVUQOF06, distance: 2.8606(14) Å, Figure 2c). The second triazole interacts with the coformer through symmetrical halogen bonds by N(3)···I(1) contacts, resulting in the formation of zig-zag chains.

In the 3D packing, the fluconazole molecules are found in rows, while the difluorobenzene rings are packed alternatively for two consecutive APIs and with the coformer in the voids as shown in the perspective view through the *a-*axis (Figure 2d). Table S2 contains a list of the geometric features of all hydrogen bonds.

#### 3.2.3 **(ITZ)_2_**·DITFB Cocrystal

**Figure 3:**
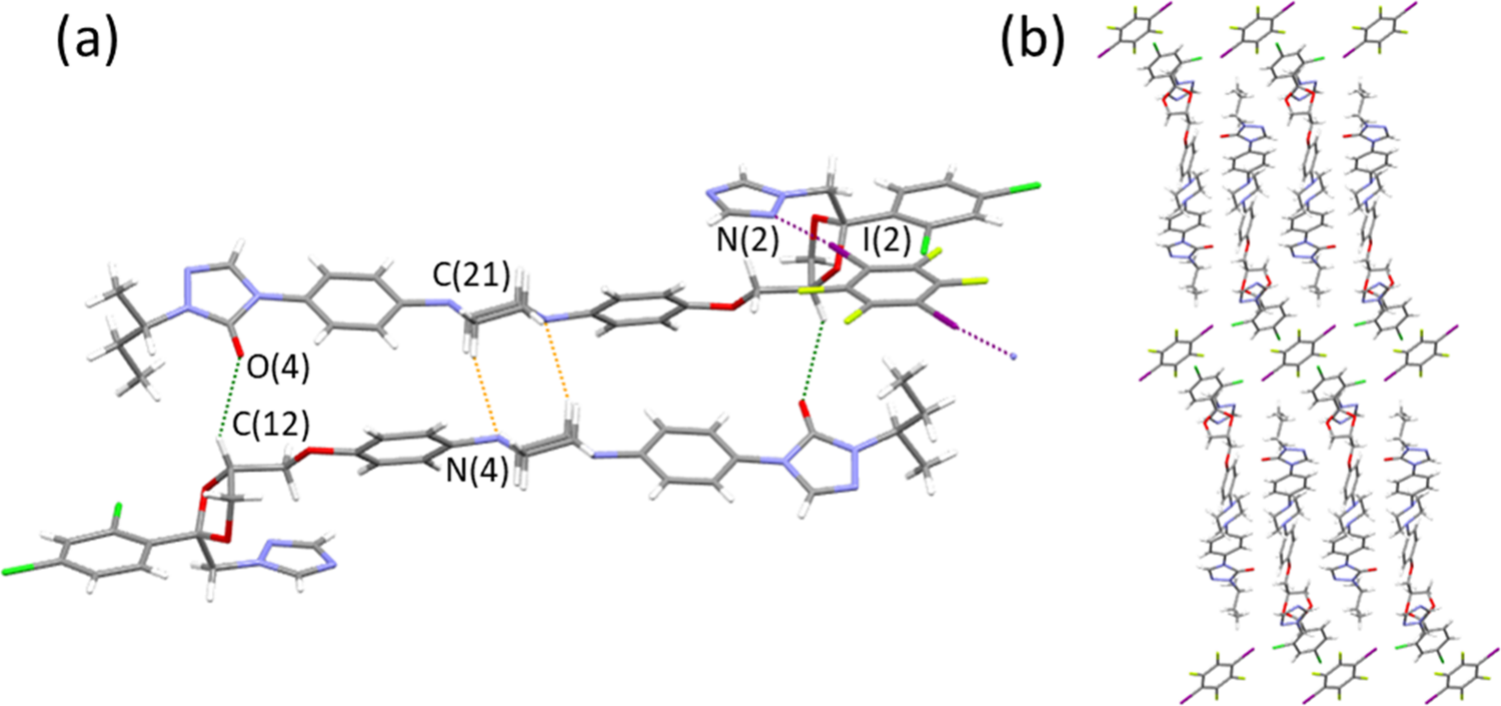
(a) **(ITZ)**_**2**_**·DITFB** cocrystal with N···I contact (in purple) and some intermolecular H-bonds (in green and orange) and (b) perspective view through the *a-*axis.

To date, only two crystal structures corresponding to **ITZ** cocrystals have been reported.^21,22,24^ This is likely due to the size, flexibility, and low solubility of this molecule. For the two reported crystal structures, hydrogen bonds are responsible for the assembly among the antifungal compound and the coformer in a sandwich mode. Herein, as far as the authors’ knowledge extends, **(ITZ)**_**2**_**·DITFB** is the first cocrystal of **ITZ** containing strong and symmetric HaB interactions. This new cocrystal crystallizes in the triclinic space group *P*1̅ containing a molecule of **ITZ** and a half molecule of **DITFB** in the asymmetric unit, which results in a 2:1 stoichiometry. In this structure, symmetrical Ha-bonds among the triazole moiety and **DITFB** were established through the N(2)···I(1) interactions, but unlike in **ITZ** cocrystals with succinic or terephthalic acid, herein the coformer is not found between two **ITZ** molecules (Figure 3a). Hydrogen bonds are also found among antifungal molecules through different interactions as, for instance, by the carbonyl group from the triazolone ring and the CH from the dioxolane ring through C=O(4)···H-C(12), between triazolone···triazole rings by N(8)···H-C(10) interactions, among piperazine rings through N(4)···H-C(21), or by fluorine atoms from the **DITFB** and the methylene groups by F(2)···H-C(8) interactions. All of them are collected in Table S3. As a result, pillars of **ITZ** molecules, in which the conazole rings are connected through the H-bonds, are related through the HaB established with the halogenated coformer besides additional F···H contacts (Figure 3b). The Ha-bond distances and angles found for the cocrystals are summarized in Table 2. Furthermore, as a measure of HaB strength, the normalized contacts (Nc) have also been included. Nc was calculated as the ratio of the observed interatomic distance with respect to the sum of the van der Waals atomic radii or Pauling ionic radii.^46,47^ The lower the NC value the stronger and well-developed the HaB is.

**Table 2:** Ha-Bond Lengths, Normalized Contacts (NC), Angles, and Energies per HaB from DFT Calculations Found in the New Cocrystals. NC were calculated using the van der Waal’s radii for atoms (*I* = 1.98 Å, *N* = 1.55 Å)^a^

cocrystal	I···A	d(I···A) (Å)	NC	<(C–I···A) (°)	Δ*E* (kcal/mol)
**VZ·DITFB**	I(2)···N(3)	2.869(7)	0.813	175.0(3)	–8.1
**(FLZ)**_**2**_**·DITFB**	I(1)···N(3)	2.8706(2)	0.813	177.82(1)	–6.5
**(ITZ)**_**2**_**·DITFB**	I(1)···N(2)	2.9205(7)	0.827	175.17(1)	–7.0

a A: acceptor.

### 3.3 Stability of Cocrystals

The thermal behavior of the new cocrystals compared to their precursors was studied to determine their thermal stability (see Figure S3). Most of the new cocrystals showed melting points lower that their former precursors. Only the **(FLZ)**_**2**_**·DITFB** resulted to have a melting point higher than fluconazole or the coformer (Table 3).

**Table 3:** Thermal Events for the Selected Conazoles, **DITFB**, and New Solid Forms

compound	*T*_peak_ (°C)	compound	*T*_peak_ (°C)
**DITFB**	108–110		
**VZ**	131.3	**VZ·DITFB**	96.0
**FLZ** (form II)	138.2	**(FLZ)**_**2**_**·DITFB**	149.6
**ITZ** (form I)	168.1	**(ITZ)**_**2**_**·DITFB**	145.1
		**ITZ·(DITFB)**_**2**_	121.6

### 3.4 DFT Calculations

The theoretical study is devoted to the energetic analysis of the intermolecular halogen-bonded synthons described above that are established between the three antifungal drugs and the ditopic halogen bond (HaB) donor molecule. Moreover, we have also evaluated the intramolecular H-bonds and π-stacking interactions in **VZ**, **FLZ**, and **ITZ** that are important to rationalize the conformation adopted in the cocrystals.

**Figure 4:**
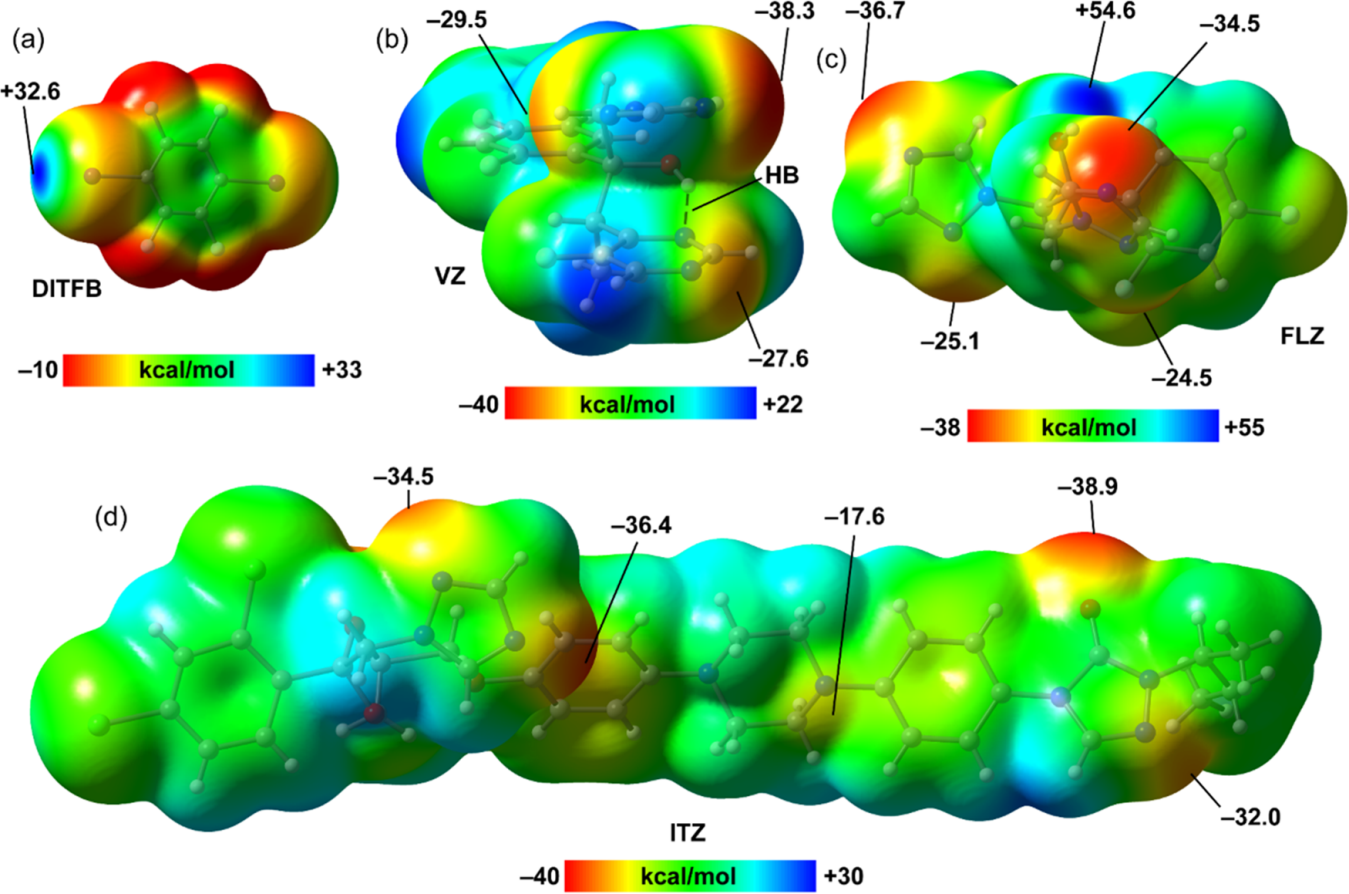
MEP surfaces of the coformers of **DIFTB** (a), **VZ** (b), **FLZ** (c), and **ITZ** (d) at the PBE0-D3/def2-TZVP level of theory (density isovalue 0.001 a.u.). The energies are given in kcal/mol.

Primarily, the MEP surfaces of all coformers have been analyzed to explore the relative HaB acceptor ability of the different N-atoms present in the fluconazole (**FLZ**), voriconazole (**VZ**), and itraconazole (**ITZ**) skeletons. The MEP surfaces are represented in Figure 4, showing that in compound **DITFB**, the MEP maxima are located at the iodine σ-holes (+32.6 kcal/mol), thus confirming the strong electrophilicity of this molecule. In compound **FLZ**, the MEP values at the N-atoms of the five-membered triazole ring are more negative than those at the pyrimidine ring, thus revealing that the triazole ring is a better halogen bond acceptor than the pyrimidine ring. Moreover, one of the N-atoms of the latter is not available to form HaBs because it is forming an intramolecular OH···N H-bond (see Figure 1b). The MEP surfaces of the three **FLZ**, **VZ**, and **ITZ** molecules evidence that the N-atom of the triazole that is located between the C-atoms is the most nucleophilic, with MEP values that range from −34.5 to −38.3 kcal/mol, in agreement with the HaBs observed in the X-ray structures of all cocrystals. In the case of **ITZ**, the MEP minimum is located at the O-atom of the five-membered ring (−38.9 kcal/mol). The MEP value at the sp^3^ N-atom piperazine ring is significantly less nucleophilic (−17.6 kcal/mol).

**Figure 5:**
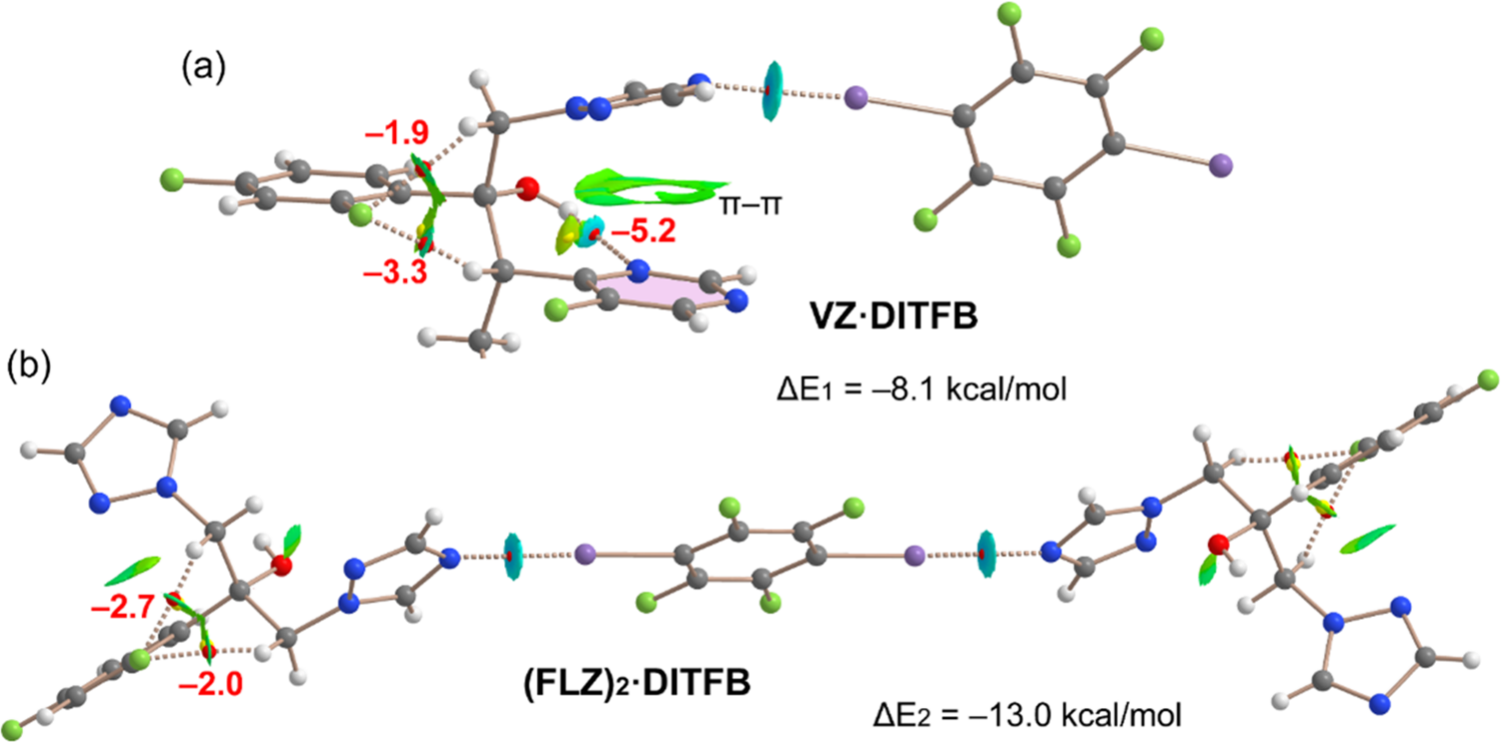
QTAIM/NCIPlot analysis of intra- and intermolecular bonds and ring CPs (red and yellow spheres, respectively), bond paths, and RDG isosurfaces of the Ha-bonded dimer of **VZ**·**DITFB** (a) and trimer **(FLZ)**_**2**_·**DITFB** (b). The individual association energies of the intramolecular H-bonds are indicated using a red font next to the bond CPs.

In order to characterize the halogen-bonding assemblies of the cocrystals, we have used a combination of QTAIM and NCIplot methods. The formation energies of the assemblies have been computed using the supramolecular approach. The combined QTAIM/NCIPlot analysis of the **VZ**·**DITFB** and **(FLZ)**_**2**_·**DITFB** cocrystals (Figure 5) shows that each I···N halogen bond is characterized by a bond critical point (CP, represented by a red sphere) and bond path (dashed bond) connecting the I and N-atoms. Moreover, a blue reduced density gradient (RDG) isosurface also emerges upon complexation, coincident with the location of the bond CP. The strength of each halogen bond is −8.1 kcal/mol in **VZ**·**DITFB** and −6.5 kcal/mol in **(FLZ)**_**2**_·**DITFB**, thus confirming the moderately strong nature of the HaB contacts, in line with the blue color of the NCIplot isosurface. Each HaB in **(FLZ)**_**2**_·**DITFB** is weaker than in **VZ**·**DITFB** likely due to anti-cooperativity effects. That is, the halogen-bonding ability of the I-atom in **DITFB** weakens upon the formation of a HaB in the opposite side of the molecule. Such effects have been recently demonstrated by Bedeković et al.^48^ in cocrystals of perfluorinated iodobenzenes with pyridines. In addition, Cinĉić et al.^49^ have also analyzed anti-cooperativity effects in three-centered halogen bonds with bifurcated acceptors present in molecular crystals, cocrystals and salts. An additional effect that explains the different strengths of the HaBs in **(FLZ)**_**2**_·**DITFB** and **VZ**·**DITFB** cocrystals is that the MEP value at the interacting N-atom of **VZ** is more negative than that of **FLZ** (see Figure 4). We have also analyzed some intramolecular interactions in both compounds and evaluated the strength of the intramolecular hydrogen bonds (HBs) by using the approximation of Espinosa et al.^50^ (using the Vr energy predictor). The QTAIM/NCIplot confirms the existence of the OH···N intramolecular H-bond in **VZ**·**DITFB** cocrystals characterized by a bond CP, bond path, and blue RDG isosurface. The strength of this intramolecular H-bond is −5.2 kcal/mol. Interestingly, the combined QTAIM/NCIplot analysis also reveals the existence of two additional CH···F bonds that further stabilize the conformation observed in the solid state (−5.2 kcal/mol for both contacts). Finally, an extended green RDG isosurface is observed between both aromatic rings, evidencing the formation of a π–π stacking interaction. In the **(FLZ)**_**2**_·**DITFB** cocrystal the CH···F contacts are also observed with similar energies (−4.7 kcal/mol for both contacts).

**Figure 6:**
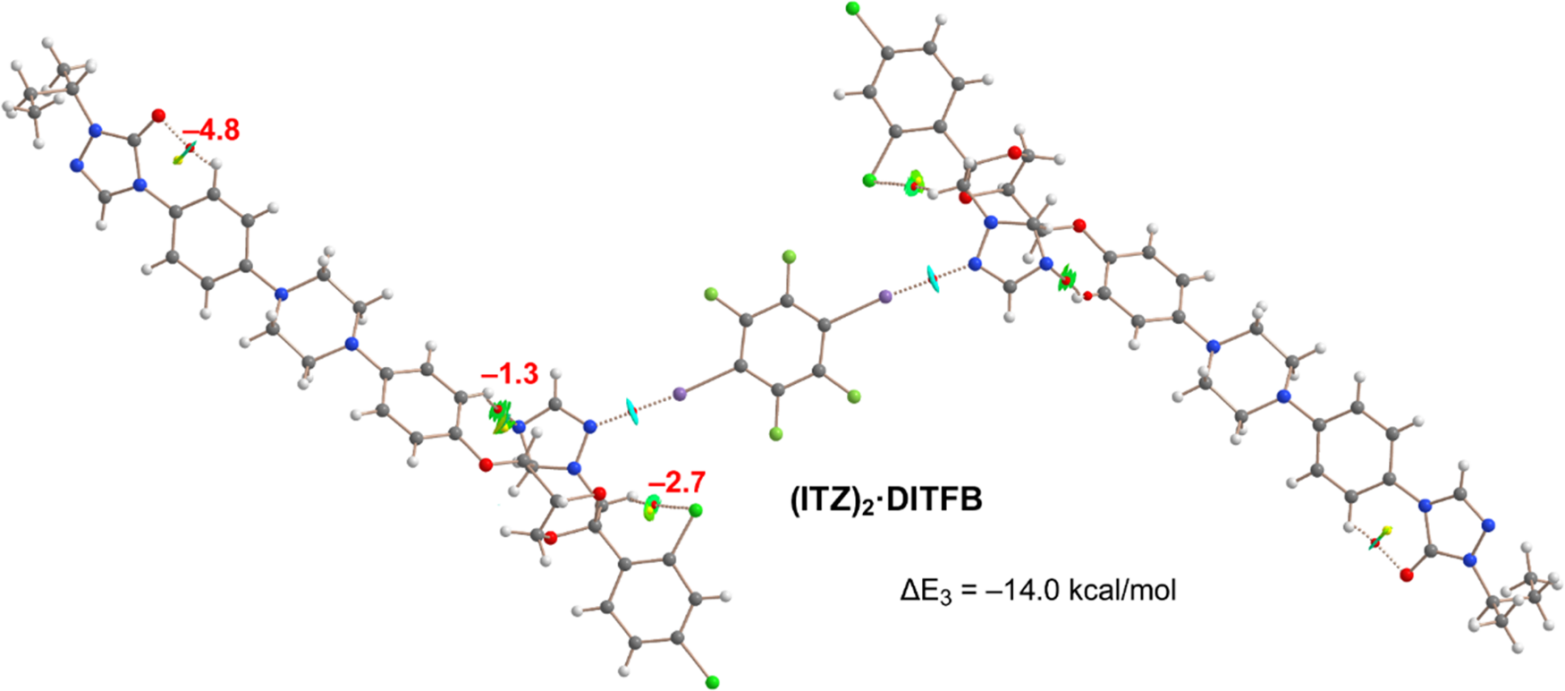
QTAIM/NCIPlot analysis of intra- and intermolecular bonds and ring CPs (red and yellow spheres, respectively), bond paths and RDG isosurfaces of the trimeric assembly of **(ITZ)**_**2**_·**DITFB**. The individual association energies of the H-bonds are indicated using a red font next to the bond CPs.

The QTAIM/NCIplot analysis of the **(ITZ)**_**2**_·**DITFB** cocrystal is depicted in Figure 6. The HaBs are characterized by bond CPs, bond paths, and RDG isosurfaces connecting the N and I-atoms, and the strength of each HaB is −7.0 kcal/mol similar to that of **(FLZ)**_**2**_·**DITFB** and weaker than that of **VZ**·**DITFB** due to utilization of both σ-holes of **DITFB**. Three intramolecular H-bonds are observed in **ITZ** influencing its conformation, which are CH···N(triazole), CH···Cl(dichlorophenyl), and CH···O(triazolone). The CH···O H-bond is the strongest one (−4.8 kcal/mol) in line with the large and negative MEP value at the O-atom of the triazolone ring (see Figure 4).

## 4 Conclusions

New cocrystals of three antifungal drugs have been synthesized and characterized. For the three APIs, these are the first halogen-bonded cocrystals described. In all of them, the structures consist of I···N(triazole) halogen-bonded molecular complexes, which confirms that the triazole is a reliable Ha-bond acceptor. For **VZ** and **FLZ**, intramolecular H-bonds are described, while for the novel cocrystal of **ITZ**, self-assembly through hydrogen-bond interactions avoids the expected sandwich packing observed in other previously described hydrogen-bonded crystal structures based on this conazole. DFT calculations in combination with QTAIM and NCIplot analyses have been used to characterize the HaBs established between the different APIs and the coformer. They revealed that the strength of the HaBs range from −6.5 kcal/mol in **(FLZ)**_**2**_·**DITFB** to −8.1 kcal/mol in **VZ**·**DITFB**, thus confirming the strong nature of these contacts and their importance in the formation of the cocrystals. We have also evaluated the strength of the intramolecular H-bonds that influence the conformation of the antifungal drugs in the cocrystals. These interactions are weaker than the HaBs. We expect that the results reported herein may inspire not only researchers working in the cocrystallization of active pharmaceutical ingredients, but also theoreticians and those working in crystal engineering and supramolecular chemistry.

## Supporting Information Available

Additional data and figures including FTIR spectra of conazoles and the new multicomponent solid forms, hydrogen bonds for **VZ·DITFB, (FLZ)**_**2**_**·DITFB** and **(ITZ)**_**2**_**·DITFB**, and TGA–DSC of conazoles and the new cocrystals (PDF)
