# Supplementary material for: Cocrystallization of Antifungal Compounds Mediated by Halogen Bonding
Source: Cryst Growth Des. 2023 Mar 8;23(4):2932–40. doi: 10.1021/acs.cgd.3c00067 (PMC10080713; doi:10.1021/acs.cgd.3c00067)
Supplement: Supplementary file 1 — cg3c00067_si_001.pdf [file cg3c00067_si_001.pdf]

Supporting Information

Cocrystallization of Antifungal Compounds mediated by Halogen bonding

Mónica Benito[†, *], *Antonio Frontera*[‡*], *Elies Molins*[†]

[†]Institut de Ciència de Materials de Barcelona (ICMAB-CSIC), Campus UAB, 08193 Bellaterra, Spain

[‡]Departament de Química, Universitat de les Illes Balears, Ctra. Valldemosa km 7.5, E-07122 Palma de Mallorca, Spain

TABLE OF CONTENTS

**Figure S1**	FTIR spectra of conazoles and the new multicomponent solid forms.
**Figure S2**	Proposed Ha-bond interactions in **ITZ·(DITFB)$_2$**.
**Table S1**	Hydrogen bonds for **VZ·DITFB** [Å and °].
**Table S2**	Hydrogen bonds for **(FLZ)$_2$·DITFB** [Å and °].
**Table S3**	Hydrogen bonds for **(ITZ)$_2$·DITFB** [Å and °].
**Figure S3**	TGA (solid lines) – DSC (dashed lines) of conazoles and the new cocrystals.

Figure S1. FTIR spectra of conazoles and the new multicomponent solid forms.

a)

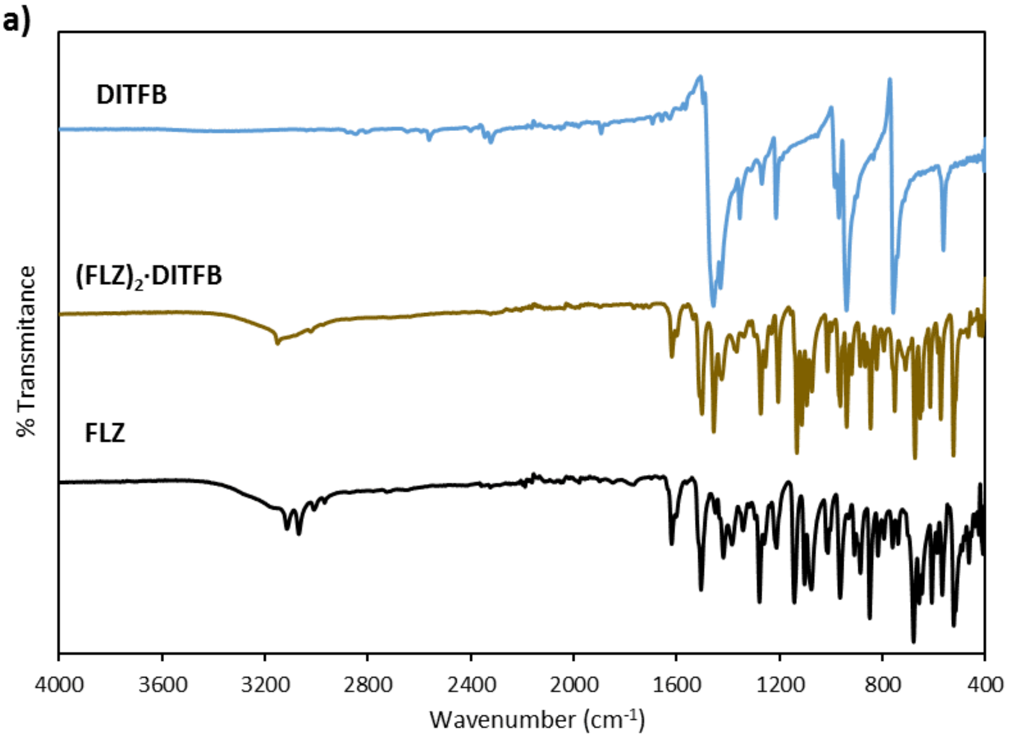

b)

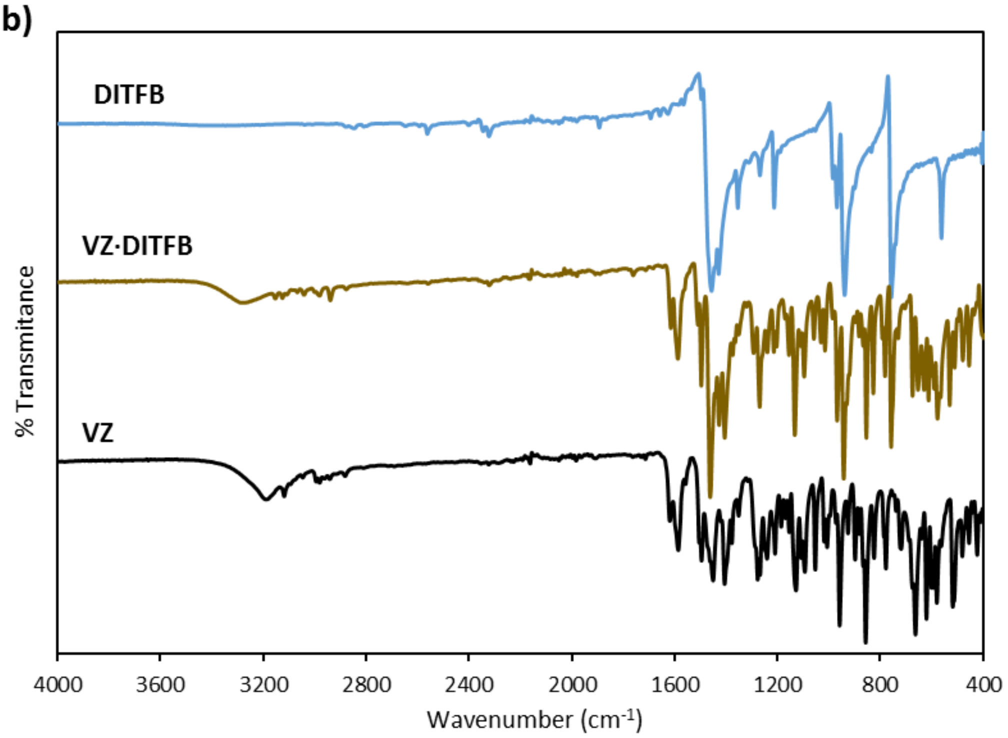

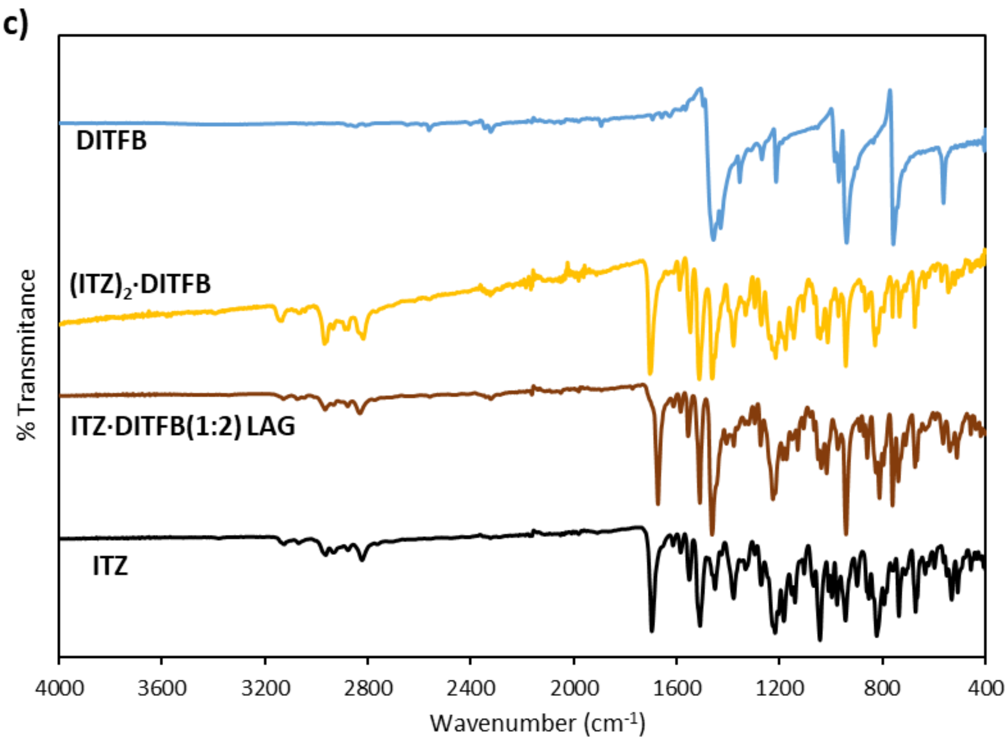

Figure S2. Proposed Ha-bond interactions in **ITZ·(DITFB)$_2$**.

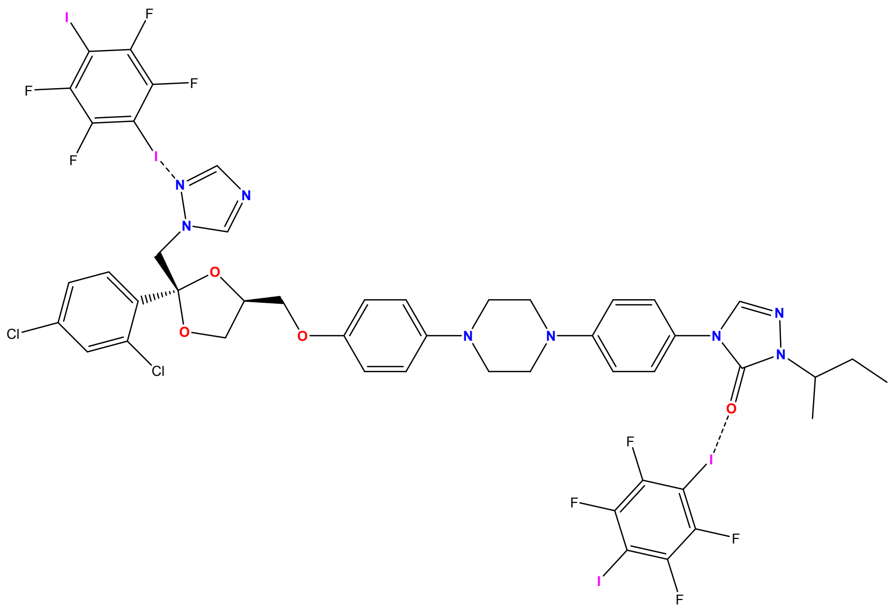

Table S1. Hydrogen bonds for **VZ·DITFB** [Å and °].

D-H···A	d(D-H)	d(H···A)	d(D···A)	<(DHA)
O(1)-H(1)···N(2)#1	0.82	2.71	3.228(9)	122.7
O(1)-H(1)···N(4)	0.82	2.07	2.784(8)	145.6
C(1)-H(1B)···F(2)	0.97	2.46	3.085(8)	122.2
C(1)-H(1B)···O(1)#2	0.97	2.65	3.538(10)	152.2
C(3)-H(3)···F(2)	0.98	2.23	2.914(9)	125.9
C(7)-H(7)···N(5)#3	0.93	2.58	3.329(10)	137.9
C(9)-H(9)···N(1)#4	0.93	2.68	3.440(11)	140.0
C(10)-H(10)···I(2)#5	0.93	3.29	4.141(9)	153.9
C(15)-H(15)···F(3)#6	0.93	2.38	3.197(10)	146.0

Symmetry transformations used to generate equivalent atoms:

#1 x,y-1,z #2 x,y+1,z #3 -x+1,y+1/2,-z+1

#4 -x+2,y-1/2,-z+1 #5 -x+1,y-1/2,-z+1 #6 -x+2,y+1/2,-z

Table S2. Hydrogen bonds for **(FLZ)$_2$·DITFB** [Å and °].

D-H···A	d(D-H)	d(H···A)	d(D···A)	<(DHA)
O(1)-H(1)···N(6)#2	0.78(3)	2.02(3)	2.773(2)	162(3)
C(8)-H(8A)···F(1)	0.97	2.43	3.030(2)	120.0
C(8)-H(8B)···F(2)#3	0.97	2.47	3.340(3)	149.0
C(9)-H(9)···F(1)#4	0.93	2.61	3.260(3)	127.4
C(9)-H(9)···F(2)#5	0.93	2.59	3.295(3)	132.8
C(11)-H(11A)···F(1)	0.97	2.40	3.005(2)	119.7
C(11)-H(11A)···N(2)#4	0.97	2.55	3.451(2)	153.9
C(11)-H(11B)···N(2)	0.97	2.63	3.199(2)	117.6
C(13)-H(13)···F(3)#6	0.93	2.43	3.317(2)	158.4

Symmetry transformations used to generate equivalent atoms:
#1 -x,-y+1,-z+2 #2 -x+2,-y,-z+1 #3 -x+1,-y,-z
#4 -x+2,-y+1,-z+1 #5 x,y+1,z+1 #6 x+1,y,z

Table S3. Hydrogen bonds for **(ITZ)$_2$·DITFB** [Å and °].

D-H···A	d(D-H)	d(H···A)	d(D···A)	<(DHA)
C(8)-H(8A)···Cl(1)	0.97	2.80	3.432(9)	123.5
C(8)-H(8B)···F(2)#2	0.97	2.54	3.462(9)	159.2
C(10)-H(10)···N(8)#3	0.93	2.59	3.353(12)	140.3
C(11)-H(11A)···O(3)	0.97	2.19	2.717(16)	113.0
C(12)-H(12)···O(4)#4	0.98	2.62	3.377(14)	134.4
C(13)-H(13B)···N(1)	0.97	2.73	3.447(11)	130.9
C(26)-H(26)···O(4)	0.93	2.29	2.913(9)	124.3
C(34)-H(34A)···I(1)#5	0.97	3.31	4.244(10)	161.5

Symmetry transformations used to generate equivalent atoms:

#1 -x+2,-y,-z+2 #2 -x+2,-y+1,-z+2 #3 -x,-y+2,-z+1

#4 -x+1,-y+1,-z+1 #5 -x,-y+1,-z+1

Figure S3. TGA (solid lines) - DSC (dashed lines) of conazoles and the new cocrystals.

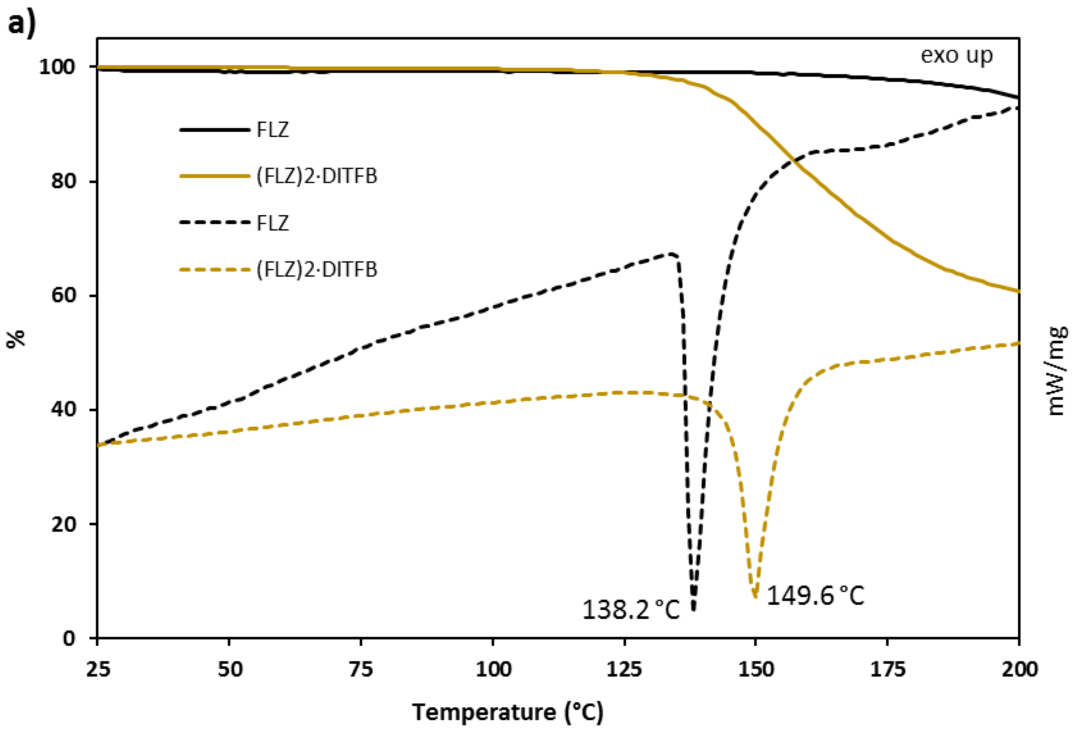

b)

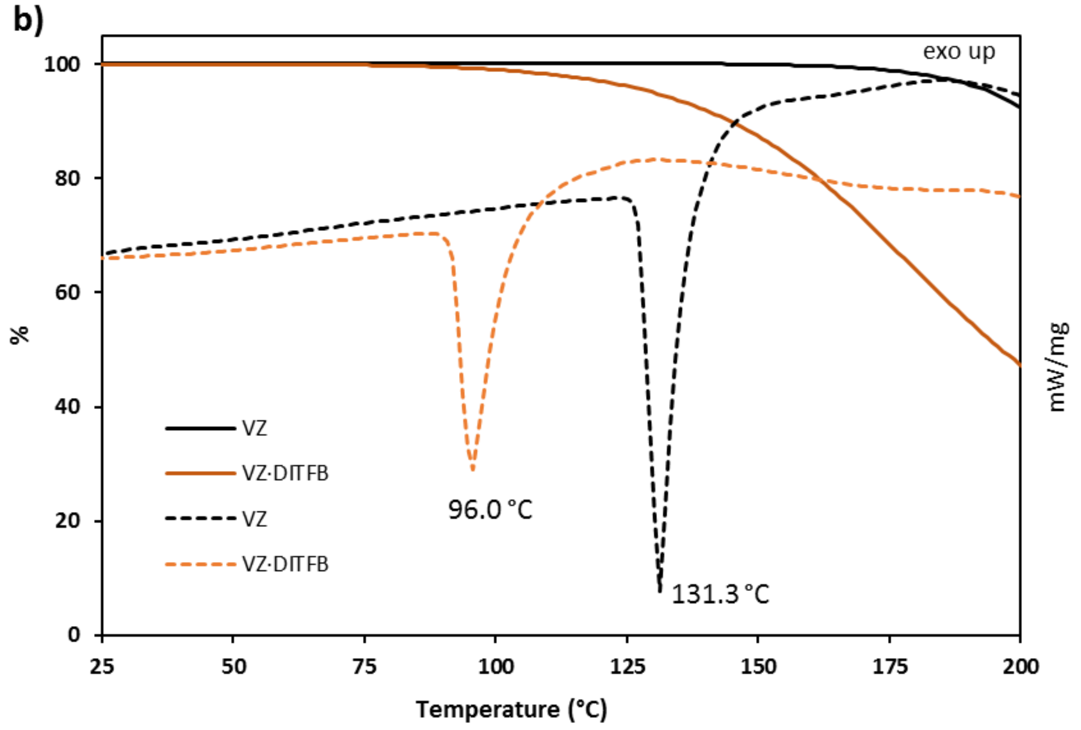

exo up
100
80
60
40
20
0
%
mW/mg
VZ
VZ·DITFB
VZ
VZ·DITFB
96.0 °C
131.3 °C
25
50
75
100
125
150
175
200
Temperature (°C)

c)

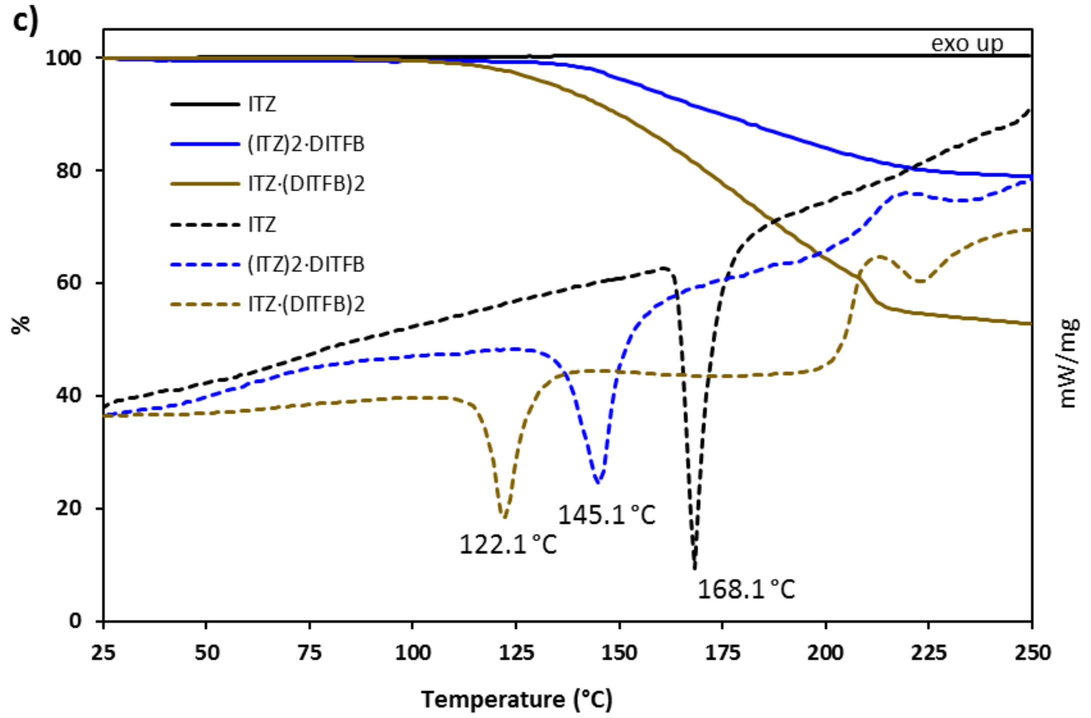

exo up
100
80
60
40
20
0
%
mW/mg
ITZ
(ITZ)2·DITFB
ITZ·(DITFB)2
ITZ
(ITZ)2·DITFB
ITZ·(DITFB)2
122.1 °C
145.1 °C
168.1 °C
25
50
75
100
125
150
175
200
225
250
Temperature (°C)
